# Performance of ACR TI-RADS and the Bethesda System in Predicting Risk of Malignancy in Thyroid Nodules at a Large Children’s Hospital and a Comprehensive Review of the Pediatric Literature

**DOI:** 10.3390/cancers15153975

**Published:** 2023-08-04

**Authors:** Jennifer R. Hess, Dane C. Van Tassel, Charles E. Runyan, Zachary Morrison, Alexandra M. Walsh, Kristian T. Schafernak

**Affiliations:** 1 Center for Cancer and Blood Disorders, Phoenix Children’s Hospital, Phoenix, AZ 85016, USA; jhess2@phoenixchildrens.com (J.R.H.); awalsh@phoenixchildrens.com (A.M.W.); 2 Department of Radiology, Phoenix Children’s Hospital, Phoenix, AZ 85016, USA; dvantassel1@phoenixchildrens.com; 3 Department of Radiology, Valleywise Hospital, Phoenix, AZ 85008, USA; charles_runyan@dmgaz.org; 4 Creighton Radiology Residency, Creighton University, Phoenix, AZ 85012, USA; ztm67829@creighton.edu; 5 Division of Pathology, Laboratory Medicine, Phoenix Children’s Hospital, Phoenix, AZ 85016, USA

**Keywords:** thyroid, nodule, pediatric, ultrasound, TI-RADS, FNA, Bethesda, molecular, thyroid cancer

## Abstract

**Simple Summary:**

Children are not little adults, when it comes to many things, especially in medicine. Sometimes, a new radiology or pathology test is developed for use in adults, and only later are pediatric applications considered—perhaps the disease being tested for is more common in adults. In this study, we aim to understand differences between adults and children when it comes to how we test for thyroid cancer. Thyroid nodules are much more common in adults, but they are much more likely to be malignant in children. Ultrasound is typically the first test when a patient has a thyroid nodule, and radiologists have developed risk stratification systems to try and determine who can be safely followed clinically and with repeat ultrasound, versus those who need to proceed to a second test, fine-needle aspiration biopsy, in which cells are removed from the thyroid and examined under a microscope, sometimes with molecular testing.

**Abstract:**

While thyroid nodules are less common in children than in adults, they are more frequently malignant. However, pediatric data are scarce regarding the performance characteristics of imaging and cytopathology classification systems validated to predict the risk of malignancy (ROM) in adults and select those patients who require fine-needle aspiration (FNA) and possibly surgical resection. We retrospectively reviewed the electronic medical records of all patients 18 years of age or younger who underwent thyroid FNA at our institution from 1 July 2015 to 31 May 2022. Based on surgical follow-up from 74 of the 208 FNA cases, we determined the ROM for the American College of Radiology Thyroid Imaging Reporting and Data System (ACR TI-RADS) ultrasound risk stratification system and The Bethesda System for Reporting Thyroid Cytopathology and added our results to those of pediatric cohorts from other institutions already published in the literature. We found the following ROMs for 1458 cases using ACR TI-RADS (TR): TR1. Benign: 2.2%, TR2. Not Suspicious: 9.3%, TR3. Mildly Suspicious: 16.6%, TR4. Moderately Suspicious: 27.0%, and TR5. Highly Suspicious 76.5%; and for 5911 cases using the Bethesda system: Bethesda I. Unsatisfactory: 16.8%, Bethesda II. Benign: 7.2%, Bethesda III: Atypia of Undetermined Significance: 29.6%, Bethesda IV. Follicular Neoplasm: 42.3%, Bethesda V. Suspicious for Malignancy: 90.8%, and Bethesda VI. Malignant: 98.8%. We conclude that ACR TI-RADS levels imply higher ROMs for the pediatric population than the corresponding suggested ROMs for adults, and, in order to avoid missing malignancies, we should consider modifying or altogether abandoning size cutoffs for recommending FNA in children and adolescents whose thyroid glands are smaller than those of adults. The Bethesda categories also imply higher ROMs for pediatric patients compared to adults.

## 1. Introduction

### 1.1. General Overview

Thyroid nodules are more common in adults compared to the pediatric population, but a much higher proportion of pediatric thyroid nodules are malignant. More specifically, thyroid nodules are present in 20–76% of adults, with prevalence increasing with age [1], whereas they are found in 0.2–5% of children and 13% of adolescents [2]. Certain subgroups of pediatric patients, however, (those with genetic risk factors (*APC*-associated polyposis, Carney complex, *DICER1* syndrome, *PTEN* hamartoma tumor syndrome and Werner syndrome) [3], radiation exposure, iodine deficiency, autoimmune thyroid disease, and goiter) are particularly likely to develop them [2]. Compared to adults, in whom only about 5% of thyroid nodules are malignant, 22–26% of pediatric thyroid nodules are malignant [3,4]. Pediatric thyroid carcinomas are also more likely to present with extrathyroidal extension [5] and regional lymph node and distant metastases (frequently, pulmonary) and carry a higher risk of recurrence [6], though children are “much less likely to die from disease...than are adults” [3].

Recommendations for the evaluation and management of pediatric thyroid nodules have historically been extrapolated from adult guidelines [7], though this approach ignores differences in clinical presentation, pathophysiology, and long-term outcomes, what might constitute appropriate therapy for an adult with thyroid cancer could be considered overly aggressive for a child [3]. Furthermore, it remains unclear whether we can use tools like the Bethesda System for Reporting Thyroid Cytopathology [8] and the Thyroid Imaging Reporting and Data System (TI-RADS) [9] to accurately predict risk of malignancy for the pediatric thyroid nodule since they were validated using predominantly adult data (when sources of validation data were even specified).

### 1.2. Toward the Successful Development of a Standardized Way of Reporting FNA Results

Before the Bethesda system, institutions and sometimes individual pathologists used their own nomenclature for reporting thyroid FNA results. Lack of a standardized approach complicated comparison of results and clinical management decision-making since there was no agreement with respect to terminology, number of categories, and their predictive value. To work toward uniformity and consensus, in October 2007, the National Cancer Institute (NCI) hosted “The NCI Thyroid Fine-Needle Aspiration State of the Science Conference”, a two-day live meeting, in Bethesda, MD. Preparations had begun 18 months earlier with the formation of a steering committee and several working committees tasked with reviewing the literature regarding various aspects of thyroid fine-needle aspiration (FNA), as well as a website to facilitate online discussion from 1 May to 15 December 2007. A summarization [10,11] of the discussions and conclusions (committee reports were also published [11,12,13,14,15,16]) formed the basis of a 2010 atlas, The Bethesda System for Reporting Thyroid Cytopathology [8], as a means for the cytopathologist to communicate clearly to the referring physician an interpretation that is “succinct, unambiguous, and clinically useful” [8], to essentially select those patients who should undergo surgical intervention and those who can be safely followed clinically and sonographically. The Bethesda system employs six diagnostic categories, each with an implied risk of malignancy (ROM) and recommended clinical management: **Bethesda I. Nondiagnostic or Unsatisfactory, Bethesda II. Benign, Bethesda III. Atypia of Undetermined Significance (AUS) or Follicular Lesion of Undetermined Significance (FLUS), Bethesda IV. Follicular Neoplasm (FN) or Suspicious for a Follicular Neoplasm (SFN), Bethesda V. Suspicious for Malignancy (SFM), and Bethesda VI. Malignant**. ROM for Bethesda II. Benign lesions is 0–3% and 97–99% for Bethesda VI. Malignant lesions. The indeterminate categories (III, IV, and V) were initially quoted to harbor ROMs of ~5–15%, 15–30%, and 60–75% [8], respectively; however, as above, those ROMs were presumably derived from predominantly adult data. Based on the limited data available, the American Thyroid Association (ATA) Guidelines Task Force on Pediatric Thyroid Cancer reported in their 2015 management guidelines for children with thyroid nodules and differentiated thyroid cancer (DTC) that indeterminate categories accounted for ~35% of pediatric FNAs, and children with Bethesda III or IV lesions actually faced much higher ROMs of 28% and 58%, respectively. As a result, “the task force recommends definitive surgery (lobectomy plus isthmusectomy) for indeterminate FNA in children”, whereas repeat FNA was an option for adults in the 2009 adult guidelines [3].

### 1.3. Building on BI-RADS: The Proliferation of TI-RADS and Many Other Ultrasound Risk Stratification Systems

**Figure 1:**
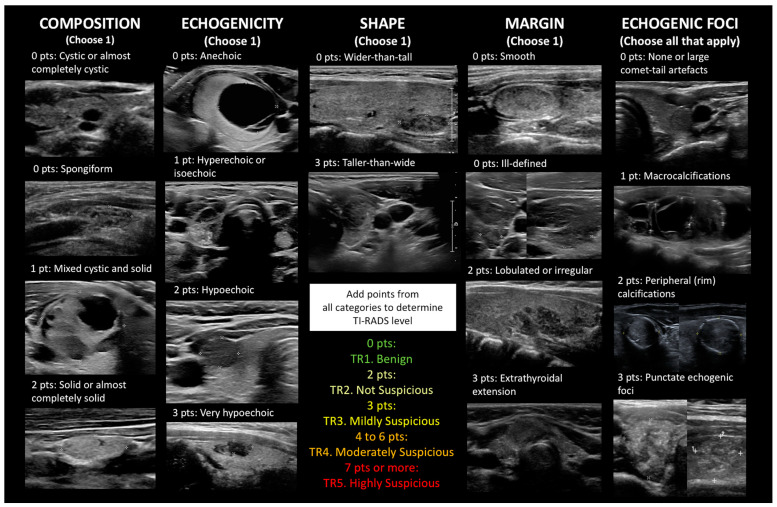
Ultrasound images (nearly all from our study population) illustrating various features of thyroid nodules and their point values by ACR TI-RADS category. The sum of the point value for each of the first four categories plus the point value of all features present in the fifth category (echogenic foci) determines the ACR TI-RADS level.

Then, in 2009, and following the success of the American College of Radiology (ACR) Breast Imaging Reporting and Data System (BI-RADS) concept, Horvath et al. [9] in Chile were the first to propose TI-RADS as a standardized ultrasound characterization and reporting data system of thyroid lesions for clinical management, basically, to select those patients who should undergo FNA. Subsequently (as well as earlier), a number of different TI-RADS or other point- or pattern-based risk stratification systems have been proposed by various groups and professional societies, such as the ones by Park et al. (2009) [17]; Kwak TI-RADS (2011) [18]; Russ et al., sometimes referred to as French TIRADS (2011) [19]; the British Thyroid Association classification (2014) [20]; the ATA grading system (2016) [21]; Korean or K TI-RADS (2016) [22]; the American Association of Clinical Endocrinologists/American College of Endocrinology/Associazione Medici Endocrinologi (AACE/ACE/AME) grading system (2016) [23]; ACR TI-RADS (2017), for which initial efforts began in 2012 and led to the publication of a lexicon in 2015 [24,25,26]; European Thyroid Association EU-TIRADS (2017) [27]; and Chinese or C-TIRADS (2020) [28]. They all fulfill important need-selecting cases for FNA according to ROM to maximize benefit and minimize cost since performing FNA on all thyroid nodules is not a rational approach [9]—in contrast to the Bethesda system, none of the different ultrasound classifications have been widely adopted worldwide [29]. With respect to ACR TI-RADS, it is point-based rather than pattern-based because a subset of nodules (some of which are malignant) may not be classifiable with the latter method (as an example, using ATA, up to 4% of nodules are unclassifiable) [21]. Points are assigned for the features of a given nodule in five different categories: composition (the presence/proportion of soft tissue and fluid), echogenicity (relative to the surrounding thyroid tissue), shape (the ratio of the anteroposterior diameter relative to the horizontal diameter when measured in the transverse plane), margins (the border or interface of the nodule with the adjacent tissue), and (presence/type of) echogenic foci. Some of the features are weighted differently to reflect their overall contribution to ROM. Figure 1 depicts examples of the various sonographic features for each category, and nearly all images were taken from our study population. Maximal size is also measured. The points are added together to determine the ACR TI-RADS level: 0 points = **TR1. Benign**, (1 to) 2 points = **TR2. Not Suspicious**, 3 points = **TR3. Mildly Suspicious**, 4 to 6 points = **TR4. Moderately Suspicious**, and 7 or more points = **TR5. Highly Suspicious**. Recommendations for FNA or follow-up ultrasound are based on the ACR TI-RADS level and, at higher TI-RADS levels, the size of the nodule. The recommendation for a TR1 or TR2 nodule is “no FNA”. For TR3, FNA is recommended if the nodule is ≥2.5 cm and follow-up imaging if it is ≥1.5 cm. As the TR level increases, the size threshold for FNA or follow-up imaging decreases: FNA at ≥1.5 cm and sonographic follow-up at ≥1.0 cm for TR4 and FNA at ≥1.0 cm and sonographic follow-up at ≥0.5 cm for TR5 [24,25,26]. The suggested RON for adults is as follows: <2% for TR1 and TR2, 2–5% for TR3, 5–20% for TR4, and >20% for TR5 [30].

### 1.4. Why Is This Study Needed?

At our own institution, in 2015, when we began offering rapid on-site evaluation (ROSE) of adequacy by a pathologist, we immediately started using the Bethesda system for reporting thyroid FNA results. We only recently began consistently using ACR TI-RADS for reporting thyroid ultrasound results, though we have done all this despite real knowledge gaps with respect to the performance characteristics and roles of ACR TI-RADS and the Bethesda system in managing children and adolescents with thyroid nodules compared to the mature body of literature on adults.

Filling these gaps is particularly important given that 1. thyroid carcinoma is the most common carcinoma occurring in the pediatric population [31] and the 4th most common cancer in adolescents [32]; 2. the incidence of pediatric thyroid carcinoma worldwide is on the rise, possibly due to multiple lifestyle (diet and obesity) and environmental factors (ionizing radiation, pollution, iodine deficiency, and polybromurate and heavy metal exposure) and increasing autoimmune disease [33,34]; and 3. major treatment advances over the past several decades have converted childhood cancer from a death sentence into a curable disease for the majority of patients [35,36], but accompanying improved survival is the unintended and unfortunate consequence of therapy-related neoplasms, particularly thyroid carcinoma in those who had received therapeutic radiation to the head, neck, or upper thorax [3,37,38,39]. Furthermore, the young thyroid appears particularly sensitive to the DNA damage induced by radiation therapy [7,38,40,41], so this is an issue not only for those who develop sporadic or syndromic thyroid carcinoma but also for the growing number of childhood cancer survivors.

Herein, we contribute our own experience at a large freestanding children’s hospital to the literature and review all of the previously published pediatric cohorts from other institutions.

## 2. Materials and Methods

Following approval by the Phoenix Children’s Hospital Institutional Review Board, we retrospectively reviewed the electronic medical records of all patients who underwent thyroid FNA with ROSE for adequacy during a nearly 7-year period (from 1 July 2015–31 May 2022). All patients had a thyroid US within 6 months of the FNA. The start date of the study period corresponded with the arrival of a pathologist experienced in thyroid cytopathology (K.T.S.); prior to their arrival, thyroid core-needle biopsy was largely performed in addition to or instead of FNA, without immediate adequacy assessment for either procedure (fully one-third of the earlier cases had been unsatisfactory) or use of the Bethesda system. During the study period, there were 223 thyroid FNA cases. In total, 15 were excluded because patient age was greater than 18 years (the older patients were primarily childhood cancer/bone marrow transplant survivors seen in our longitudinal survivor clinics); thus, a total of 208 cases were included. Informed consent was waived, and the study was HIPAA-compliant.

As the ultrasound findings were not uniformly clinically reported using the ACR TI-RADS system during the study period, all cases were retrospectively and independently reviewed by a single board-certified pediatric radiologist (D.C.V.T.), whose practice encompassed both pediatric and adult radiology. The radiologist was masked to cytology and histology results.

FNA results were reported using the Bethesda system by one of three pathologists practicing cytopathology.

Histology was considered the gold standard, and the diagnosis of follicular thyroid carcinoma (FTC) or papillary thyroid carcinoma (PTC) was based on the criteria outlined in the World Health Organization Classification of Tumours, 5th edition: Endocrine and Neuroendocrine Tumours [42], with pathologic “TNM” staging following the 8th edition of the American Joint Committee on Cancer Cancer Staging Manual [43].

Standard descriptive summaries of the data were prepared using percentages for categorical variables and means for numerical variables. Sensitivity, specificity, positive predictive value (PPV), and negative predictive value (NPV) were calculated for each TI-RADS level, Bethesda category, and combined TI-RADS and Bethesda score. The area under the curve (AUC) was determined using the JROCFIT JavaScript program for calculating receiver operating characteristic (ROC) curves (available from http://www.jrocfit.org, accessed on 11 May 2023).

## 3. Results

### 3.1. Clinical Characteristics from Our Institution

The 208 thyroid FNA cases came from 142 patients ranging in age from 3.1–18.8 years (mean, 13.6), with 112 (78.9%) female patients (age range, 3.1–18.8 years; mean, 13.5) and 30 (21.1%) male patients (age range, 8.1–18.6 years; mean, 14.2). Ninety-four patients underwent only one thyroid FNA, whereas 32 had two thyroid FNAs, 11 had three, 3 had four, and 1 had five thyroid FNAs. The vast majority of patients with multiple thyroid FNAs had different nodules sampled simultaneously, whereas five patients had sequential FNAs of the same nodules over time. Four FNAs were of the thyroid bed after lobectomy or total thyroidectomy to assess whether there was recurrence of tumor or regrowth of/remnant benign thyroid tissue. When cellular material is obtained, thyroid bed FNA has high sensitivity and specificity for the diagnosis of recurrent malignancy [44].

### 3.2. ACR TI-RADS Results from Our Institution

Table 1 shows the distribution, mean nodule size, and mean TI-RADS points for all cases by TI-RADS level and specific categorical features. ROM using histology as the gold standard is provided for total TI-RADS points, TI-RADS levels, and categorical features. Briefly, nearly one-half (100 or 48.1%) of all nodules were **TR4. Moderately Suspicious**. Fifty-six or 26.9% of cases were **TR3. Mildly Suspicious**, whereas **TR5. Highly Suspicious** accounted for 31 or 14.9% of cases. Appropriately, as this retrospective study was based on patients who underwent FNA, the least common levels were **TR1. Benign** and **TR2. Not Suspicious** at 3.8% and 6.3% of cases, respectively. The most frequent feature for each category was solid for composition (81.3% of cases), hypoechoic for echogenicity (53.8%), taller-than-wide for shape (91.3%), smooth for margins (52.4%), and none/large comet-tail artifacts for echogenic foci (81.7%). The features for each category that were associated with the highest mean TI-RADS point total were solid for composition (4.7 total points), very hypoechoic for echogenicity (6.7), wider-than-tall for shape (7.3), lobulated/irregular for margins (6.5; note that none of our cases had extrathyroidal extension), and punctate echogenic foci for echogenic foci (7.5). ROM for **TR1. Benign** was 0%, 20% for **TR2. Not Suspicious**, 41.2% for **TR3. Mildly Suspicious**, 37.5% for **TR4. Moderately Suspicious**, and 72.2% for **TR5. Highly Suspicious**. The one malignant TR2 case was a minimally invasive FTC, which, on ultrasound, was a solitary mixed cystic and solid, isoechoic, wider-than-tall 3.8 cm nodule, with smooth margins and no echogenic foci causing discomfort to the patient when eating solid foods. FNA cytopathology was Bethesda III, and lobectomy revealed a 3.5 cm well-circumscribed cystic and solid tumor. In general, the FTCs had lower TI-RADS points/levels than the PTCs (mean TIRADS level of 3.2 for FTC versus 4.3 for PTC); therefore, a low TI-RADS point total or level did not entirely exclude malignancy, particularly FTC. The features in each category that were associated with the highest ROM were solid for composition (53.3%), very hypoechoic for echogenicity (50%), taller-than-wide for shape (50%), ill-defined for margins (54.2%), and punctate echogenic foci for echogenic foci (77.3%).

### 3.3. ACR TI-RADS Results from the Pediatric Literature, including Our Cases

Table 2 summarizes the ROM for the cases of ours that had histologic follow-up in the context of previously published pediatric cohorts [5,6,45,46,47,48,49,50,51,52], which will be discussed further below.

### 3.4. Bethesda Results and Cyto/Histo Correlation from the Pediatric Literature, Including Our Cases

**Figure 2:**
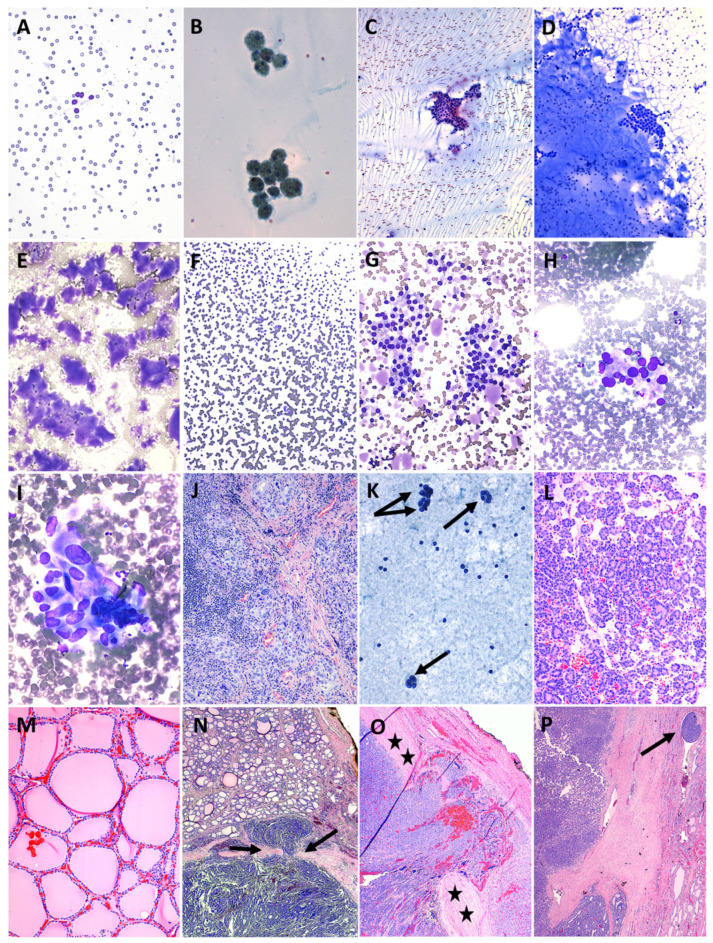
Cytology/histology images for Bethesda categories I–IV. **Bethesda I. Unsatisfactory**: (**A**) Despite four FNA passes, this solid and cystic nodule yielded blood almost exclusively, with only very rare follicular cells (a cluster of 5 cells is present in the center of the image); typically, a minimum of six groups of well-visualized follicular cells with at least 10 cells per group is required to be considered adequate for diagnosis (Diff-Quik, 200×). **Bethesda II. Benign**: (**B**) An exception to the above is a colloid nodule; note the presence of abundant colloid and groups of macrophages containing golden-brown hemosiderin pigment (Papanicolaou, 200×). (**C**) Note the waves of thin watery colloid and benign follicular cells. Benign nodules usually have a high colloid/cell ratio (Papanicolaou, 100×). (**D**) This Diff-Quik-stained smear from the same patient in (**C**) has thicker colloid at the left and bottom of the image, with the thinner colloid in the upper right cracking as it air-dries (100×). (**E**) Occasionally the colloid is so dense that it forms chips (Diff-Quik, 100×). (**F**) Sometimes the colloid is so thin and watery that it is almost invisible, but its positive charge reduces the “zeta potential” that normally repels the negatively charged red blood cells from one another so that they stack up or aggregate as “rouleaux” (Diff-Quik, 100×). (**G**) This smear contains blood, colloid, and small, uniform, darkly staining and cytologically banal follicular cells (Diff-Quik, 200×). **Bethesda III. Atypia of Undetermined Significance**: (**H**,**I**) This FNA of a nodule from a 13-year-old girl on methimazole for Graves’ disease showed rather striking cytologic atypia in the form of nuclear enlargement, anisonucleosis, and irregular nuclear contours (Diff-Quik, 200× and 400×). Such features can be concerning for (and actually may represent) PTC but caution should be exercised to not overinterpret the findings in this particular context. (**J**) Histologic follow-up revealed similar nuclear atypia and focally dense chronic inflammation but there was no evidence of neoplasm or malignancy (H&E, 100×). **Bethesda IV. Follicular Neoplasm**: (**K**) Papanicolaou-stained smear (200×) from a 9-year-old girl with a 5.4 cm thyroid nodule showing microfollicles (arrows). FNAs of follicular neoplasms are frequently bloody, as in this case. (**L**) Histology (H&E, 100×) corresponding to the FNA shown in (**K**); note the high cellularity and microfollicles containing relatively scant colloid and how this contrasts to the normal appearance of the contralateral lobe in (**M**) (also H&E, 100×). The distinction between follicular thyroid adenoma and follicular thyroid carcinoma cannot be made on cytologic grounds and instead can only be determined after excision; invasion through the tumor capsule or vascular invasion outside the capsule are the criteria for carcinoma. In (**N**) (H&E), there is minimal invasion as follicular thyroid carcinoma “mushrooms” through the capsule (indicated by arrows); observe how different the appearance of the tumor on the bottom half of the image is compared to the adjacent involved parenchyma at the top. (**O**) (H&E) is an example of widely invasive carcinoma, where the tumor extends to the right side of the image, well beyond the thick fibrous capsule indicated by stars. (**P**) (H&E) illustrates vascular invasion by tumor (arrow) outside the capsule.

**Figure 3:**
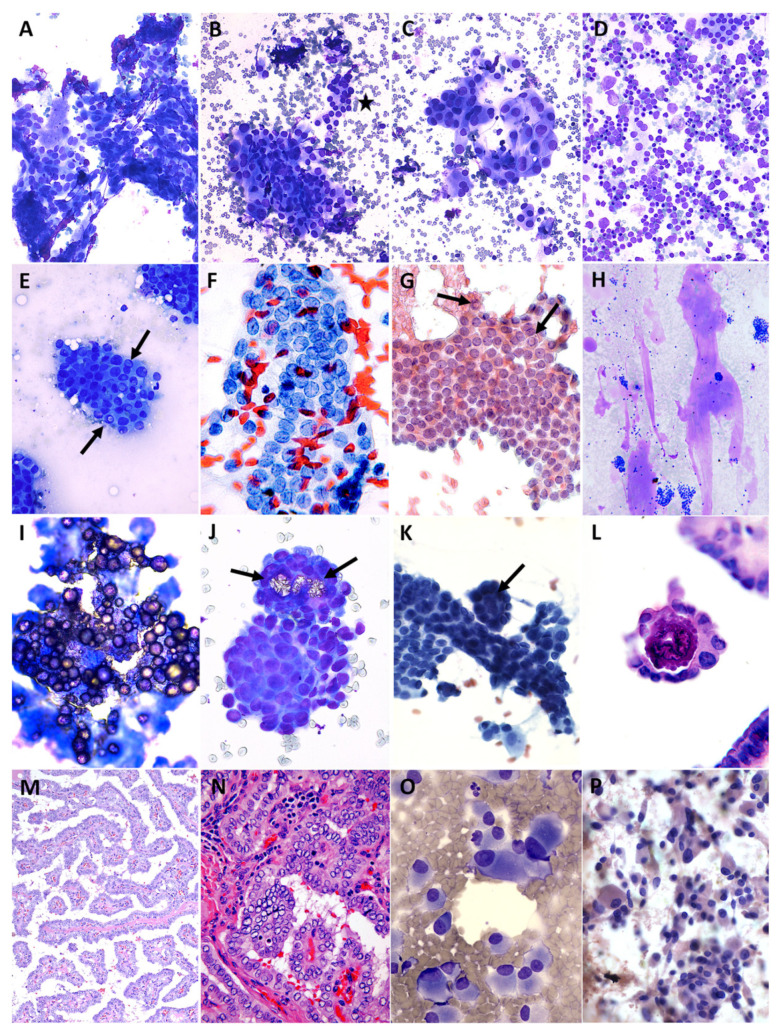
Cytology/histology images for Bethesda categories V–VI. **Bethesda V. Suspicious for Malignancy:** (**A**–**D**) (Diff-Quik) are from a 0.95 cm nodule in a 17-year-old girl with positive autoantibodies (anti-TPO and anti-thyroglobulin). We had settled on calling this suspicious for PTC because of architectural atypia with overlapping nuclei (**A**) and cytologic atypia with nuclear enlargement and variability ((**B**); compared to normal nuclei adjacent to the star) and cells with dense squamoid cytoplasm (**C**), but sent the case to an outside institution for additional expert review, where it was called AUS with atypical follicular cells and atypical lymphoid cells (**D**) with a recommendation to pursue molecular testing, which showed negative (normal) results. We communicated our extremely judicious use of this Bethesda category and persistent concern for PTC despite the consultant’s opinion and normal molecular results, and we recommended excision. PTC was confirmed histologically. **Bethesda VI. Malignant**: (**E**) Malignant aspirates tend to have high cellularity and little to no colloid, and one of the most specific features for PTC is the presence of intranuclear cytoplasmic invaginations (INCIs, a couple of which are indicated by arrows in the Diff-Quik-stained smear). (**F**) Longitudinal grooves almost make the nuclei resemble coffee beans and are a useful, albeit less specific, feature of PTC (Papanicolaou). (**G**) The chromatin in PTC tends to be pale and dusty with micronucleoli marginating to the nuclear membrane (Papanicolaou, 400×). Arrows point to INCIs that are thought to be an adaptation to increase the surface area for exchange between the nucleoplasm and cytoplasm, as nuclear pores are defective in PTC. (**H**) Occasional cases of PTC show dense colloid that smears out like bubble gum stuck to one’s shoe (Diff-Quik). (**I**) This spectacular PTC aspirate (100×) contains many psammoma bodies, which are clear, colorless, and refractile on Diff-Quik. Psammoma bodies are concentrically lamellated microcalcifications that develop as the tips of papillae in PTC undergo necrosis and the process of dystrophic calcification. (**J**) Arrows point to a few psammoma bodies in a different case of PTC (Diff-Quik, 400×). Also notice how you can see through the nuclei as they overlap in this irregular, three-dimensional structure. (**K**) A papillary structure on its side goes from upper left to lower right, whereas another one is present en face, with a psammoma body (arrow) coming towards you. (**L**) Histologic appearance of a psammoma body at the tip of a papilla in PTC (H&E). (**M**) Finger-like papillae with fibrovascular cores are seen both longitudinally as well as en face in this histology image of PTC (H&E). (**N**) This higher magnification image (H&E) of PTC depicts some of the same nuclear features (enlargement and overlap) that we see on FNAs, but the optical clarity of the nuclei (often referred to as “Orphan Annie” nuclei from the comic strip) is an artifact of formalin fixation. (**O**) (Diff-Quik) and (**P**) (Papanicolaou). Aspirate of medullary thyroid carcinoma.

**Figure 4:**
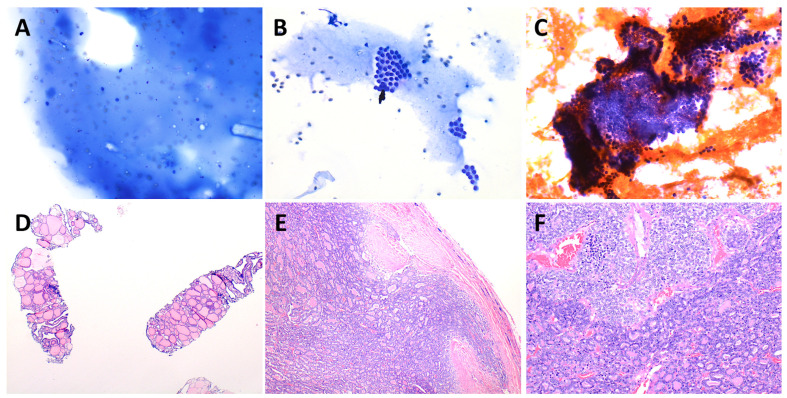
False-negative FNA and core biopsies which turned out to be PTC, likely attributable to sampling error. A 13-year-old girl with multinodular goiter underwent FNA of five separate thyroid nodules. Two were TI-RADS 4, and core biopsies of those were also taken. All were called Bethesda II. Benign: Consistent with benign follicular nodule, though, at thyroidectomy, one of the TI-RADS 4 nodules, an encapsulated 1.4 cm lesion, was actually a PTC (consensus recommendation at the multidisciplinary conference had been total thyroidectomy, as opposed to long-term surveillance with serial ultrasounds and FNA as needed). On retrospective review of the FNA/core biopsy slides, we determined this major discrepancy was not an interpretive error and instead might have been due to sampling. The FNA smears from this particular nodule were variably bloody but contained at least a moderate amount of colloid accompanied by cytologically banal follicular cells ((**A**,**B**), Diff-Quik, 200×); a single questionable focus was present on the Papanicolaou-stained smear ((**C**), 200×), though it was obscured by blood. Multiple tissue cores ((**D**), H&E, 20×) showed completely benign thyroid tissue with variably sized follicles, in contrast to the appearance of the lesion at resection, which was an invasive encapsulated follicular variant of PTC with focal invasion into but not through the tumor capsule ((**E**), H&E, 40×) and small zones of cells with nuclear crowding and chromatin clearing typical of PTC (at top of image (**F**), H&E, 100×), without papillary configuration or psammoma bodies. The diagnosis of PTC was further corroborated by multifocal membrane positivity for HBME-1. Given the coexistence of multiple other adenomatous and colloid nodules, some showing papillary hyperplasia, we sent for molecular testing, which revealed a *DICER1* hotspot mutation.

**Figure 5:**
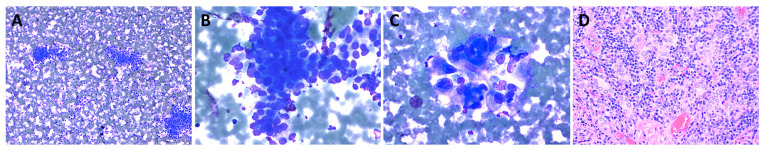
False-positive FNA case which was actually Hashimoto thyroiditis, a diagnostic pitfall. A 10-year-old boy with an history of hypothyroidism underwent FNA of an ACR TI-RADS 4 left thyroid nodule (solid and ill-defined, with punctate echogenic foci). The Diff-Quik-stained smears contained a few three-dimensional papillary-like structures ((**A**), 100×) with enlarged “see-through” overlapping nuclei ((**B**), 400×) in a background of chronic inflammation. Some cells had angulated nuclear profiles ((**C**), 400×). This FNA was called Bethesda VI. Malignant: Papillary thyroid carcinoma, and preoperative lymph node mapping demonstrated a suspicious lymph node adjacent to the trachea. Gross examination revealed a 5 cm mass, though, on microscopic examination of the entire thyroidectomy specimen, there was only severe chronic lymphocytic thyroiditis with focal reactive nuclear clearing ((**D**), H&E, 200×) and no PTCs in the gland or any of the lymph nodes examined.

Cytology results were correlated with histology results when available and are shown toward the bottom of Table 3, which summarizes the frequency, ROM, and risk of neoplasm (RON) when available by Bethesda category for our patients as well as published pediatric cohorts [6,53,54,55,56,57,58,59,60,61,62,63,64,65,66,67,68,69,70,71,72,73,74,75,76,77,78,79]. In addition, representative cytology and histology images are presented in Figure 2 and Figure 3. Briefly, **Bethesda category I. Unsatisfactory** accounted for 7.7% of cases in our cohort, with an implied ROM of 40% (RON, 40%), including two PTCs. **Bethesda II. Benign** represented 56.7% of our cases, with an ROM of 4.8% (RON, 19.0%) based on a single false-negative PTC case likely attributable to sampling error, which is presented in Figure 4. An interpretation of **Bethesda III. AUS** was rendered in 21.6% of our cases, with an ROM of 27.3% (three FTCs, three PTCs; RON, 56.9%). **Bethesda IV. FN** and **Bethesda V. SFM** were uncommon at 2.4% and 1.4% of cases, respectively, both with an ROM and RON of 100% (three FTC and three PTC for Bethesda IV, and two PTC for Bethesda V; the PTC cases had follicular and/or solid growth patterns). Finally, 10.1% of cases were diagnosed as **Bethesda VI. Malignant**, with an ROM of 94.7% (18 PTCs and one false-positive case of Hashimoto thyroiditis that had concerning clinical, sonographic and cytologic features—see Figure 5). Concordance was determined for Bethesda categories II–VI using the approach of Heider et al. [68]: cases were considered concordant if they were cytologically benign and histologically non-neoplastic; cytologically atypical and histologically neoplastic or malignant; cytologically follicular neoplasm/suspicious for follicular neoplasm and histologically neoplastic; or cytologically suspicious for malignancy or malignant and histologically malignant. Concordance was as follows: Bethesda II, 81.0%; Bethesda III, 59.1%; Bethesda IV, 100%; Bethesda V, 100%; and Bethesda VI, 94.7%; with an overall concordance of 79.7%.

### 3.5. The Potential Value of a Combined Score That Incorporates TI-RADS and Bethesda

**Figure 6:**
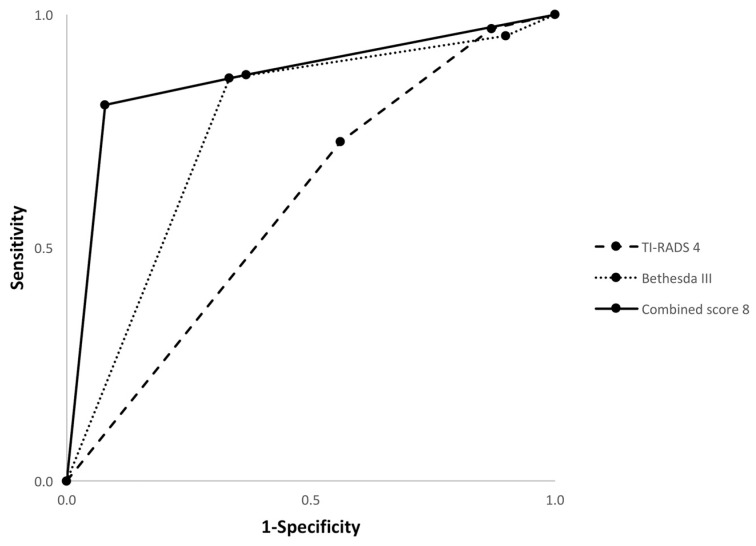
ROC curves for optimal TI-RADS, Bethesda, and combined score cutoffs. For TI-RADS, the AUC is 0.66, standard error is 0.077, and 95% confidence interval is 0.54–0.78. For Bethesda, the AUC is 0.83, standard error is 0.005, and 95% confidence interval is 0.74–0.93. For the combined score, the AUC is 0.89, standard error is 0.077, and 95% confidence interval is 0.81–0.97.

Combining the TI-RADS level and Bethesda category (excluding the unsatisfactory Bethesda I cases) into a single score (e.g., TI-RADS 3 and Bethesda III = combined score of 6) showed a sharp cutoff between 7 and 8, whereby cases with a combined score of 7 or less had a ROM ranging from 0 to 17.6%, whereas cases with a combined score of 8 or more had a ROM ranging from 71.4 to 100%. This is shown in the lower right of Table 2. ROC curves were developed for TI-RADS level, Bethesda category, and combined score, and they are superimposed in Figure 6. Accuracy measures are provided in Table 4.

## 4. Discussion

### 4.1. Multiple Ultrasound Systems Have Been Applied to Pediatric Thyroid Nodules

Although none of the different ultrasound risk stratification systems have been widely adopted worldwide like the Bethesda system has been for reporting thyroid FNA results [29], in pediatrics, the greatest number of general studies (to our knowledge 11, including our own) have involved ACR TI-RADS [5,6,45,46,47,48,49,50,51,52]. Seven pediatric studies have examined the ATA grading system [5,6,50,61,80,81,82], and a smaller number of studies have looked at EU-TIRADS [5,50,52,74], K TI-RADS [5,50,83] (one compared the diagnostic performance of the 2021 K TI-RADS criteria to those of 2016 K TI-RADS) [83], Kwak TI-RADS [46,81], or AACE/ACE/AME [5] in children and adolescents. Some of these studies used the same set of pediatric cases to compare the performance of different ultrasound systems against each other [5,6,46,50,52,81]; for example, Kim et al. [5] retrospectively analyzed a total of 277 thyroid nodules from 221 pediatric patients using ACR TI-RADS, ATA, K TI-RADS, EU TI-RADS, and AACE/ACE/AME. Kim et al., in a different article, did a systematic review and meta-analysis of ACR TI-RADS and ATA in pediatric thyroid nodules [30], and Piccardo et al. compared ACR TI-RADS, ATA, and EU-TIRADS in children with thyroid nodules and history of therapeutic radiation for a non-thyroidal primary malignancy [84]. Finally, a few studies have evaluated individual sonographic characteristics [85,86,87].

### 4.2. Performance of ACR TI-RADS in Pediatrics

Overall, when combining our results with those already in the pediatric literature (Table 2), ACR **TR1. Benign** carries with it an ROM of 2.2%, with an 9.3% ROM for TR2. Not Suspicious, 16.6% for **TR3. Mildly Suspicious**, 27.0% for **TR4. Moderately Suspicious**, and 76.5% for **TR5. Highly Suspicious**. It is important to point out the way that ROM was determined since it varied by study. For our cohort, we considered surgical/histologic follow-up to be the gold standard for outcome (this was how the ROM was determined in almost all of the cases with follow-up in Table 3 for the Bethesda system), though the majority of other studies used a combination of FNA results or surgical follow-up, sometimes in conjunction with clinical follow-up/sonographic stability or decrease in size, or increased activity on nuclear medicine scan. As the follow-up method varied by study, ROM for each category was similarly variable between studies, ranging from 0 to 25% for TR1 and TR2, 0–42.9% for TR3, 0–68.4% for TR4, and 38–100% for TR5.

### 4.3. Comparison of ACR TI-RADS to Other Ultrasound Systems in Pediatrics

How does ACR TI-RADS perform relative to the other ultrasound systems in pediatrics? Shapira-Zaltsberg et al. [46] compared ACR TI-RADS to Kwak TI-RADS and found no significant difference in diagnostic performance (AUC for ACR, 0.74 versus 0.72 for Kwak), though interrater agreement was superior with ACR (*p* < 0.001). Using malignant histology as the gold standard, Ahmad et al. [6] compared ATA with ACR TI-RADS, and, whereas ATA had a higher sensitivity of 84.6% (TI-RADS was 76.9%), ACR TI-RADS had higher specificity (71.4% versus 9.5%), accuracy (73.5% versus 38.2%), PPV (62.5% versus 36.7%), and NPV (83.3% versus 50.0%). According to the ATA criteria, FNA would have been recommended for 114 of 138 nodules, though 2 nodules with histologically proven malignancy would have been missed; adhering to ACR TI-RADS recommendations based on TR level and size would have resulted in FNA of only 32 nodules, but 3 nodules with histologically proven malignancy would have been missed. Naturally, finding the “sweet spot” for a decision threshold means carefully balancing the benefit of early cancer detection against the risk of missing malignancy as well as subjecting children and adolescents with benign nodules (and their families) to unnecessary procedures typically involving anesthesia, at least in our institution. The authors also examined how adapting ACR TI-RADS could improve performance characteristics of the test, specifically reducing the size threshold for biopsy (FNA if ≥1.5 cm for TR3, ≥1.0 cm for TR4, and any technically feasible size for TR5)—what they call “PED TI-RADS”—or removing nodule size altogether from the recommendations, instead performing biopsies of nodules of any size with a TR level of 3 or higher or TR4 or higher. They found that PED TI-RADS, like ATA, would have missed two histologically proven malignant nodules, although the number of FNAs recommended would have dropped by 50, from 114 to 64. Using an ACR TI-RADS level of 4 as the cutoff, irrespective of nodule size, would have further reduced the number of FNAs recommended to 43, but four histologically proven malignancies would have been missed. Dropping the cutoff to TR3, irrespective of nodule size, increased sensitivity to 100%, and 20 FNAs could have been avoided compared to ATA. In contrast to the study by Lim-Dunham et al. [45], in which there was only one false-negative case (a TR1 malignant nodule), Richman et al. found in their cohort of 404 pediatric thyroid nodules that by following ACR TI-RADS recommendations, 17 (22.1%) of 77 malignant nodules would have been missed at initial presentation, 9 would have been assigned follow-up, but the other 8 would not have been assigned follow-up [48].

Scappaticcio et al. [50] concluded that ACR TI-RADS, EU-TIRADS, K TI-RADS, and ATA all “have suboptimal performance in managing pediatric patients with thyroid nodules, with one-half of cancers without indication for FNA according to their recommendations”. They found a sensitivity of 41.7% for ACR TI-RADS and EU-TIRADS and 50.0% for K TI-RADS and ATA, a “missed malignancy rate” of 58.3% for ACR TI-RADS and EU-TIRADS and 50% for K TI-RADS and ATA, and an “unnecessary FNA prevalence” of 58.3% for ACR TI-RADS and EU-TIRADS and 76% for K TI-RADS and ATA. Their results contrasted with those of a larger study by Kim et al. [5], who, as mentioned above, applied five different ultrasound risk stratification systems to 277 pediatric thyroid nodules. They found that the diagnostic performances of all five (ACR TI-RADS, ATA, K TI-RADS, EU TI-RADS, and AACE/ACE/AME) “were acceptable in the pediatric population and were improved by applying the American College of Radiology Thyroid Imaging Reporting and Data System size cutoffs for nodules 1 cm or larger and allowing biopsy of the highest category nodules smaller than 1 cm”. Tuli et al. [52] looked at EU TI-RADS and ACR TI-RADS and found that ACR TI-RADS “performed better than EU-TIRADS as also observed in previous [adult] studies”, though 6 (23.1%) of 26 cancers would have been missed. Finally, in their 2021 systematic review and meta-analysis evaluating ACR TI-RADS and ATA in a total of 1036 pediatric thyroid nodules from eight articles, Kim et al. [30] found pooled ROMs for ACR TI-RADS to be 5.5% for TR1, 6.0% for TR2, 11.0% for TR3, 34.2% for TR4, and 59.3% for TR5, and, for ATA, 7.5% for very low suspicion pattern, 12.2% for low suspicion pattern, 34.2% for intermediate suspicion pattern, and 55.4% for high suspicion pattern. Pooled sensitivity and specificity for the two highest categories in each system were 84% and 64%, respectively, for TR4 and TR5 and 90% and 50% for intermediate and high suspicion, but specificity for TR5 alone was significantly higher (97%) compared to high suspicion (66%) (*p* = 0.02). Unnecessary biopsy rate was 62.7%, missed malignancy rate was 21.7% for ACR TI-RADS, and the authors felt that lowering cutoff size for FNA would be a reasonable option to increase test sensitivity and decrease the missed malignancy rate. They also commented that the clinical context such as family history of thyroid cancer and personal history of exposure to ionizing radiation and also the presence or absence of suspicious cervical lymph nodes should be given greater weight when it came to selecting those pediatric patients for FNA, as acknowledged in the ATA guidelines [3,30]. Piccardo et al. [84] studied pediatric patients previously treated with radiotherapy for non-thyroidal cancers—a high-risk group—and found that ACR TI-RADS, ATA, and EU TI-RADS did not indicate the need for FNA in 6 (42.9%), 7 (50%), and 8 (57.1%) of 14 histologically proven PTCs, and, in 5 cases, it was due to subcentimeter nodule size. Shapira-Zaltsberg et al. [46] had earlier and similarly concluded that adjustment of TI-RADS was necessary in pediatrics, taking into account “presence or absence of pathological-appearing lymph nodes and pediatric nodule size modification”. At least in this way, we could recognize the fact that thyroid volume in children is not the same as in adults [5].

### 4.4. Individual Sonographic Characteristics Associated with Malignancy in Pediatrics

Other studies did not look at risk stratification systems but instead focused on identifying individual sonographic characteristics that were associated with a higher ROM in children. Al Nofal et al. [85] did a systematic review and meta-analysis of 12 studies that comprised a combined total of 750 nodules. Enlarged/suspicious lymph nodes and internal calcifications had the highest likelihood ratios for malignancy (4.96 and 4.46, respectively). Richman et al., using the same cohort of 404 pediatric nodules that they used to evaluate ACR TI-RADS [48], found the highest PPVs for abnormal lymph node (77.1%), lack of smooth margin (70.7%), and speckled calcifications alone (67.2%) [86]. We should also point out that they also found substantial interobserver reliability (kappa, 0.72) for presence of absence of abnormal lymph nodes—important to consider since Cozzolino et al., in their meta-analysis of 14 studies comprising 1306 thyroid nodules in the “transition age” (mean/median age of patients included ranging from 12 to 21 years), reported the highest diagnostic odds ratio (DOR) for malignancy for the presence of suspicious lymph nodes (56.0), followed by the presence of microcalcifications (13.0), irregular margins (9.0), and a “taller-than-wide” shape (6.0) [87].

### 4.5. How Does ACR TI-RADS Perform in the Adult Setting?

With the pediatric data in mind, it is useful to briefly familiarize ourselves with how ACR TI-RADS performs in adults. A few large systematic reviews and meta-analyses have compared ACR TI-RADS to Kwak TI-RADS [88] or ATA and K TI-RADS [89] or examined inter-reader reliability [90]. Kang et al. [88] assessed 46 studies with a total of 39,085 patients and found that the highest AUC for ACR TI-RADS was 0.875 for TR5. TR5 had the highest specificity as well at 87.0% (it was 52.2% for TR4 and 23.7% for TR3), whereas the inverse was the case for sensitivity (71.0% for TR5, 94.4% for TR4, and 98.9% for TR3). DOR was 17.5 for TR4, 17.3 for TR5, and 15.3 for TR3. In total, 11 studies evaluated the diagnostic accuracies of both ACR and Kwak TI-RADS on the same sets of patients or nodules. There were no significant differences between ACR TR4 and the corresponding Kwak TR level, 4b, in terms of AUC, sensitivity, specificity, or DOR; the same was true for ACR TR5 and Kwak 4c, the highest level in that system. Li et al. [89] included 16 studies with a total of 21,882 nodules from 18,164 patients: 10 of the studies compared ACR TI-RADS and ATA, whereas 6 directly compared ACR and K TI-RADS. ACR TI-RADS had a pooled sensitivity of 89%, pooled specificity of 70%, AUC of 0.86, and DOR of 18.5. There were no significant differences between ACR TI-RADS and ATA in terms of pooled sensitivity (83% versus 87%; *p* = 0.5) or pooled specificity (69% versus 50%; *p* = 0.1), or between ACR and K TI-RADS pooled sensitivity (85% versus 91%; *p* = 0.13), but the pooled specificity of ACR TI-RADS (57%) was significantly superior to that of K TI-RADS (24%) (*p* < 0.001). The authors concluded that “ACR TI-RADS showed favorable sensitivity and moderate specificity” and that “The use of ACR TI-RADS could avoid a large number of unnecessary biopsies, although at the cost of a slight decline in sensitivity”. Li et al. [90] also looked at 13 studies comprising 5238 nodules to determine pooled inter-reader agreement for overall ACR TI-RADS classification, which was moderate, with a kappa of 0.51. In terms of inter-reader agreement for the different categories of sonographic features, composition had the highest kappa at 0.58; shape was 0.57; echogenicity was 0.50; echogenic foci was 0.44; and margin was 0.34. Ha et al. [91] applied seven society guidelines to 2000 consecutive thyroid nodules that were ≥1 cm. They found that the Korean Thyroid Association/Korean Society of Thyroid Radiology, National Comprehensive Care Network, and ATA all had significantly higher specificities than AACE/ACE/AME, ACR TI-RADS, the French Society of Endocrinology, and the Society of Radiology in Ultrasound (*p* < 0.001), but the latter had significantly higher specificities (*p* < 0.001). The unnecessary FNA biopsy rate was lowest for ACR TI-RADS at 25.3%.

### 4.6. The Application of Artificial Intelligence to Adult and Pediatric Thyroid Ultrasound

Machine learning, a subfield of artificial intelligence, has also recently been applied to thyroid ultrasound to see if it can help radiologists better predict malignancy and reduce unnecessary FNAs. Zhao et al. [92] compared a machine learning-assisted visual approach and a separate radiomics approach with ACR TI-RADS. The machine learning-assisted visual approach, based on human feature extraction and computational techniques, was developed from the consensus interpretation of two experienced radiologists in a training data set of 520 nodules regarding six ultrasound parameters (the five ACR TI-RADS categories of composition, echogenicity, shape, margins, and echogenic foci, plus maximal size), with or without five shear wave elastography (SWE) parameters (SWE-mean, SWE-min, SWE-max, SWE-SD, and SWE-ratio)—shear wave elastography imaging provided information on nodule hardness. The radiomics approach (computer-based image analysis) was developed by having two radiologists delineate regions of interest in the ultrasound and SWE images, and software was used to extract 6940 radiomics features for each region of interest in six different classes: contour/shape/textural phenotype features, histogram features, second-order textural features, filter-based features, intra-perinodular textural transition features, and co-occurrence of local anisotropic gradient orientations features. Feature reduction and selection methods were used to come up with 10 machine learning classifiers, and both approaches were optimized and applied to a validation data set of 223 nodules and then to a test data set of 106 nodules from another hospital. The machine learning-assisted ultrasound visual approach showed better diagnostic performance (AUCs, 0.900 for validation set and 0.917 for test set) than the ultrasound radiomics approach (0.789 for validation set and 0.770 for test set) or ACR TI-RADS (0.689 for validation set and 0.681 for test set). Adding SWE to ultrasound improved the AUCs for the machine learning-assisted visual approach to 0.951 for the validation set and 0.953 for the test set and decreased the unnecessary FNA rate to 4.5% in the validation set (for ACR TI-RADS, it was 30.0%) and 4.7% in the test set (compared to 37.7% for ACR TI-RADS) [92]. While their patients were predominantly adults, machine learning has also been applied to small pediatric cohorts. Radebe et al. [93] applied “random forests” (a type of machine learning method) in conjunction with interpretable rule sets to demographic, ultrasound, and biopsy data from patients under 18 years with thyroid nodules and found that their models predicted nonbenign cytology and malignant histology better than historical outcomes. Yang et al. [94] compared the overall impressions (benign versus malignant) of three independent radiologists with ACR TI-RADS and a previously developed deep learning algorithm on 139 patients 21 years or younger. Sensitivity for radiologists’ overall impressions ranged from 32.1% to 75.0% (mean, 58.3%) compared to 82.1% to 87.5% (mean, 85.1%) for ACR TI-RADS and 87.5% for the deep learning algorithm. Specificity ranged from 63.8% to 93.9% (mean, 79.9%) for radiologists’ overall impressions, compared to 47.0% to 54.2% (mean, 50.6%) for ACR TI-RADS and 36.1% for the deep learning algorithm. Thus, the deep learning algorithm had comparable sensitivity to ACR TI-RADS, though specificity was lower [94].

### 4.7. The Frequency, Risk of Malignancy and Risk of Neoplasm in the Various Bethesda System Categories in Pediatrics

Returning to thyroid FNA results, as above, Table 3 summarizes the raw, calculated, and overall data for frequency, risk of malignancy, and risk of neoplasm for published pediatric case cohorts, including our own, using the Bethesda system [6,57,58,59,60,61,62,63,64,65,66,67,68,69,70,71,72,73,74,75,76,77,78,79,80,81,82,83]. Out of a total of 5911 published cases (including our own), with surgical follow-up for 2486 cases and 2 years or greater clinical follow-up for an additional 57 and unknown clinical follow-up for 5 more cases, the **Bethesda I. Unsatisfactory** category accounted for 11.4% of cases, with an implied ROM of 16.8% (RON, 26.7%), **Bethesda II. Benign** accounted for 56.0% with a ROM of 7.2% (RON, 27.5%), **Bethesda III. AUS** accounted for 9.6% with a ROM of 29.6% (RON, 55.8%), **Bethesda IV. FN** accounted for 6.4% with a ROM of 42.3% (RON, 86.8%), **Bethesda V. SFM** accounted for 3.9% with a ROM of 90.8% (RON, 97.6%), and **Bethesda VI. Malignant** accounted for 12.7% with a ROM of 98.8% (RON, 99.7%). While we only included the 2019 to 2021 Children’s Hospital of Philadelphia cases in Baran et al. [76] to eliminate overlap with cases previously reported by Jia et al. [72], we cannot exclude the possibility of redundancy that some, if not many or even all, of the 44 FNA cases in Gallant’s retrospective consecutive case series and genomic classifier study of FNA and FFPE tissue from sequential pediatric thyroidectomies [77] had been previously reported by Wang et al. [63]. In our review of the literature to determine the ROM, we excluded cases that were called out as low-risk neoplasms, including 12 cases of non-invasive follicular thyroid neoplasm with papillary-like nuclear features (NIFTP), 5 cases of follicular tumor of uncertain malignant potential (FT-UMP), and 2 cases of well-differentiated tumor of uncertain malignant potential (WT-UMP) because their risks of recurrence or other adverse events like metastasis were extremely low [42]; they were, of course, included in the RON calculations along with follicular thyroid adenoma (FTA) and oncocytic adenoma of the thyroid (formerly known as Hürthle cell adenoma). We also excluded pediatric studies that used a classification system other than Bethesda, such as the Italian Working Group SIAPeC-IAP (Società Italiana di Anatomia Patologica e Citopatologia Diagnostica-International Academy of Pathology) classification [95,96,97,98,99,100], or did not provide sufficient granularity of data [4].

### 4.8. The Bethesda System Does, in Fact, Perform Differently in Children Compared to Adults

Several meta-analyses have been published regarding the frequency and ROM for the different Bethesda categories in adults. In 2012, Bongiovanni et al. [101] summarized 8 articles with a total of 25,445 FNA cases, 6362 (25.0%) which had histological follow-up, and the ROMs for each Bethesda category were in line with what had been published in the 1st edition of the Bethesda book [8]. In 2015, Straccia et al. [102] reviewed 51 articles that provided a total of 145,928 FNA cases, and they focused on the 4475 AUS/FLUS and 3202 FN/SFN cases, which had overall ROMs of 27% and 31%, respectively; while the ROM for the FN/SFN category was at the upper end of the range quoted in the 1st edition of the Bethesda book (15–30%), the ROM of 27% for AUS/FLUS was quite a bit higher than the approximately 5 to 15% quoted in the 1st edition [8]. This was one of the articles cited in the 2nd edition of the Bethesda book, where the ROM for AUS/FLUS was increased to ~10–30% (note that for FN/SFN, the ROM was also pushed up to 25–40%) [103], though the meta-analysis by Krauss et al. showed overlapping 95% confidence intervals for AUS/FLUS (11–23%) and FN/SFN (20–29%), leading them to suggest that these categories have similar ROMs [104]. In 2020, Vuong et al. investigated differences in resection “rate” and ROM between Western (American and European) and Asian countries, analyzing a total of 38 studies comprising 145,066 FNA cases [105]. Statistically significant differences were observed for ROM in Bethesda categories II, III, and V (the ROMs were all higher in the Asian series), accompanied by a higher frequency of Bethesda IV cases in the Western series and a higher frequency of Bethesda VI cases in the Asian series. In fact, this meta-analysis formed the basis for comparison in their separate meta-analysis of 3687 pediatric cases [106], and, although they concluded that there were no significant differences in ROM between pediatric and adult thyroid nodules for any of the Bethesda categories, their study was criticized by Cherella et al. for ignoring significantly lower resection rates for adults “potentially obscuring true differences in ROM” [107], which Vuong et al. had even pointed out in their own article for all Bethesda categories except Bethesda I [106]. Cherella et al. continued by stating that “Of course, the actual ROM lies between the values calculated among all nodules and among resected nodules [107]”, which is recognized in the 2nd edition of the Bethesda book [103]. In their published response, Vuong et al. offered an alternative metric, the overall risk of malignancy or “oROM” (the proportion of malignant nodules to all aspirated nodules), and, although they qualified their results by stating that such a metric was not used in the vast majority of existing publications about thyroid nodules and that its utility is not well-acknowledged at present, there were, in fact, significant differences (*p* < 0.001) between the oROMs they calculated for pediatric versus adult thyroid nodules for Bethesda III (21.5% versus 9.2%), Bethesda IV (36.9% versus 17.1%), Bethesda V (82.8% versus 53.9%), and Bethesda VI (91.9% versus 70.8%) [108].

### 4.9. Accounting for Bias

Indeed, it is important to be aware of bias and account for it when possible. In our study, as well as nearly all published pediatric cohorts, surgical follow-up was used to determine the ROM for the Bethesda categories (this is in contrast to the pediatric studies we reviewed on ACR TI-RADS, which used various forms of follow-up, or no follow-up at all, to determine ROM). Relying on histology at resection excluded those patients managed by clinical and sonographic follow-up and affected the diagnostic accuracy of the test by “partial verification bias”, a type of bias that occurs when “only a proportion of the study participants receive confirmation of the diagnosis by the reference standard test [109]”. This could underestimate the number of false-negative cases and overestimate the sensitivity [109]. With respect to the meta-analysis by Vuong et al. discussed above [106], Cherella et al. had commented that “Accounting for this bias is crucial when comparing ROM between pediatric and adult nodules because lower resection rates in adults lead to greater overestimation of the ROM in adults than in children” [107]. Buryk et al. also stated that “Surgical case series studies...inherently overestimate the risk of cancer” [54]. In addition, studies performed at tertiary care centers may have benign cases from the community filtered out, thereby making them subject to selection bias, in which the studied population might not be totally representative of the “real world” [54].

### 4.10. What if We Add Clinical and Sonographic Results to Bethesda Results?

Similar to our own study, some authors have combined Bethesda results with clinical and sonographic features with or without molecular results to differentiate between benign and malignant pediatric nodules [54,61,110]. Buryk et al. found significant differences between groups when it came to nodule size (larger nodules were more likely to be malignant), incidental imaging finding (incidentally discovered nodules were more likely to be benign), palpable nodule, palpable lymphadenopathy, both palpable nodule and palpable lymphadenopathy, and molecular mutation positive (all more likely to be malignant) [54]. The McGill Thyroid Nodule Score (MTNS) integrated clinical, radiologic, and pathologic findings that were associated with a higher risk of thyroid cancer in adults. In their pilot study, Canfarotta et al. adapted the McGill criteria for pediatric use, combining clinical parameters and laboratory tests such as sex, whether the nodule was palpable, its consistency on exam, thyroid-stimulating hormone (TSH) concentration, history of ionizing radiation, and family history of thyroid cancer, with the following sonographic criteria and the Bethesda category: echogenicity, increased vascularity, shape, calcifications, enlarging, abnormal lymph nodes, and size. Points were assigned and added together to reach a final score. They found significant differences (*p* < 0.05) between 36 patients with benign and 10 patients with malignant final pathology for TSH > 1.4 mIU/L, hypoechoic echogenicity, microcalcifications, and lymphadenopathy, all greater in the malignant group. A final pediatric MTNS of 11 was shared by both benign and malignant cases, but a score of 10 or lower was only seen in benign nodules, whereas a score of 12 or more was only seen in the malignant ones [61]. Creo et al. [110], in a larger study of 99 patients aged 21 years or younger (66 with benign and 33 with malignant nodules on final pathology), found that the average pediatric MTNS was 1.7 +/− 2.9 for benign nodules and 12.7 +/− 4.3 for malignant nodules. Setting the pediatric MTNS cutoff at 8 or higher provided 93.2% sensitivity and 93.1% specificity for malignancy. Adjusting it to 9 or higher resulted in a slight drop in sensitivity to 90.9%, but the specificity went up slightly to 96.6%. However, a Bethesda category of 4 or higher independently predicted malignancy with 97.7% sensitivity and 94.0% specificity. Therefore, the ability of the pediatric MTNS to predict malignancy might be largely derived from the cytology results [110]. The study by Tan et al. [111] was most similar to ours in that instead of integrating clinical and other laboratory findings, they simply added an ultrasound score for TI-RADS to a Bethesda score and determined test characteristics for TI-RADS alone, Bethesda alone, and the combined score, though they did not use ACR TI-RADS; the scores were weighted differently instead of corresponding directly to the TI-RADS level and Bethesda category, and their patients were mostly adults (average age, 45.33 +/− 12.17 years), though they did include patients as young as 12. They concluded that “The combination of high-resolution ultrasonography TI-RADS classification and US-FNAC (Bethesda classification) can improve the accuracy of malignant thyroid nodules diagnosis” [111].

### 4.11. Applying the Bethesda System to Frozen Section Diagnosis

Arnold and Nicol reported their success with applying the Bethesda system to frozen section diagnosis in children [112] and found a similar ROM to FNA cytology, though this was not something we considered at our institution.

### 4.12. Subtyping AUS by Type of Atypia or Reclassifying AUS by TI-RADS

Returning to the clinicopathologic dilemma of indeterminate FNA results, the Bethesda system [103] outlines the most common scenarios for which a Bethesda III (AUS) interpretation is appropriate: cytologic atypia; architectural atypia; cytologic and architectural atypia; Hürthle cell aspirates; atypia, not otherwise specified; and atypical lymphoid cells, ruling out lymphoma. Some authors have found that certain types of atypia carry a relatively greater or lesser ROM within the AUS category, and consistently cytologic (nuclear) atypia or a combination of cytologic (nuclear) and architectural atypia have been shown to carry a significantly higher ROM that cases with architectural atypia alone [113,114,115,116]. However, these were not pediatric studies. The most common types of atypia we encountered in our cohort were cytologic atypia (AUS-C, 16 cases), cytologic and architectural atypia (AUS-C/A, 15 cases), and architectural atypia (AUS-A, 11 cases). Eight of the AUS-C cases had histologic follow-up, with a 50% RON (including two FTAs) and a 25% ROM (two PTCs), whereas seven of the AUS-C/A cases had histologic follow-up, with a 85.7% RON (including two FTAs and one oncocytic adenoma of the thyroid) and a 42.9% ROM (two FTCs and one PTC). The RON and ROM as determined on histologic follow-up of 4 AUS-A cases was 25% (one FTC). Although the numbers are small, these findings suggest that the type of atypia may not be quite as important in pediatric thyroid FNAs compared to those of adults, though a recent pediatric study did find that nuclear atypia (but not architectural atypia) was associated with a significantly increased risk of malignancy [117]; nevertheless, more pediatric studies are needed. There may also be a role to reclassify pediatric AUS cases based on radiologic features: Arva and Deitch [55] found that pediatric AUS/FLUS cases with a low ultrasound score (although they did not use TI-RADS, they did look at echogenicity, size, vascularity, margins, calcifications, and cystic or solid (composition)) had a ROM of 11% compared to cases with a high ultrasound score, which had a ROM of 28.5%. These results were not substantiated by our cohort, though. On histologic follow-up, our TR2 or TR3 AUS cases had a ROM of 50% (3/6 cases), whereas our TR4 or TR5 AUS cases had a ROM of only 23.1% (3/13 cases). Perhaps additional pediatric studies will address the utility of looking back at the ultrasound findings using deep learning models after an indeterminate FNA result in the same way that Gild et al. did with older patients [118].

### 4.13. Why Rapid On-Site Evaluation Is Important

The major benefit of performing ROSE is the ability to provide real-time feedback to the proceduralist regarding specimen adequacy so that additional FNA passes can be obtained until adequacy is reached. ROSE has been shown to reduce the frequency of nondiagnostic specimens [119]. In our own institution, it basically drove the dramatic increase in our FNA case volume (not just thyroids) and helped us to transition away from the practice of core-needle biopsies on every thyroid case with or without FNA, whereas the interventional radiologists rapidly gained experience with the procedure. In addition, if during the procedure it appeared that an FNA was going to be inadequate or indeterminate, it allowed us to simultaneously obtain cores for histology and/or molecular testing, or if the nodule was too small or in a precarious location for core biopsy, to obtain additional passes for ThinPrep (which we quickly abandoned due to lack of success) or cell block; in some new PTC cases, we were even able to offer lymph node sampling at the same time. Multiple studies have demonstrated the higher diagnostic yield of core-needle biopsy compared to repeat FNA for Bethesda I and Bethesda III cases [120,121,122,123], and performing core-needle biopsy concurrently rather than sequentially has been more efficient for us. Like core biopsies, cell block material can also be used in Bethesda III cases for immunohistochemistry [124], with a combination of Hector Battifora mesothelial-1 (HBME-1), galectin-3, and cytokeratin 19 being the set of biomarkers mostly commonly assessed by investigators [125]. While we have occasionally found immunohistochemistry for mutant BRAF V600E protein a useful adjunct to morphology [126,127], we have not had good experience confirming *ALK* translocations or *NTRK* fusions with ALK or pan-TRK immunostains, whereas others have found immunohistochemistry to an efficient and reliable screening approach that can be followed by more expensive fluorescence in situ hybridization or RNA sequencing [128,129].

### 4.14. Limitations of the Current Study and the Pediatric Literature in General

There are other limitations of our study and in the pediatric literature in general. While the size of our cohort was, for example, larger than nearly three-quarters of the previously published pediatric studies on the Bethesda system (Table 3), there is a handful of studies with a greater number of FNA cases than we had. In addition, histologic follow-up was only available for 35.6% of our FNAs, though this limitation was common to many of the previously published studies. However, we tried to overcome these limitations by comprehensively reviewing the pediatric literature on ACR TI-RADS, the Bethesda system, and the impact of molecular testing on clinical care.

Only one attending radiologist was involved in retrospective review of the ultrasound images; retrospective study design is in it itself a limitation, but we do not have any data from our patient cohort for interobserver (or intraobserver) agreement, though, in the previous pediatric ACR TI-RADS papers, it ranged from as low as 0.37 (fair) to as high as 0.85 (very good/almost perfect) correlation; intraobserver agreement was only reported in one study and was substantial at 0.69–0.77 (Table 2). Similarly, whereas three pathologists were mainly involved in reviewing the FNA cytology, we did not look at interobserver or intraobserver agreement for the Bethesda system. For quality assurance purposes, many of the FNAs had been seen by at least one additional pathologist, and the category assigned represented a consensus diagnosis; however, there are data (though not pediatric-specific) regarding intra- and interobserver agreement using Bethesda. Intraobserver agreement has been less studied. Kuzan et al. reported substantial intraobserver agreement (kappa, 0.705) for one pathologist and moderate intraobserver agreement (kappa, 0.447) for another, though, when compared with a cytopathologist, interobserver agreement was “below the lowest acceptable limit for an overall agreement...among the three raters”, with an alpha of 0.634 [130]. Pathak et al. found substantial agreement (Fleiss’ kappa, 0.6561) between three raters of different experience levels, with higher agreement between a consultant with greater than 20 years of experience in cytopathology and a senior resident with 4 years of experience (Cohen’s kappa, 0.7517), compared to the consultant and a junior resident with 6 months of experience (Cohen’s kappa, 0.5907). They concluded that diagnostic accuracy increases with experience, and the Bethesda system “is usable by even a beginner in cytopathology” [131]. Kappa values ranged from 0.735 to 0.841 when comparing three pathologists to each other in a study by Ahmed et al. [132], and Anand et al. [133] reported a Cohen’s weighted kappa score of 0.99 among three pathologists in another single institution study; however, Słowińska-Klencka et al. found poor interobserver agreement among five experienced cytopathologists from three centers (Krippendorff’s alpha coefficient, 0.34) when reclassifying smears from indeterminate (Bethesda III, IV, and V) cases, with fair intra-center but poor inter-center agreement, with combined ROMs for Bethesda categories IV, V, and VI varying widely between centers [134]. This is not unexpected, as another study found the highest agreement between two cytopathologist “experts in thyroid FNA” for nondiagnostic/unsatisfactory (Bethesda I) and malignant (Bethesda VI) cases (100%), followed by 93.9% agreement for benign (Bethesda II) cases, 66.7% agreement for the FN/SFN (Bethesda IV) and SFM (Bethesda V) categories, and only 50% of AUS/FLUS (Bethesda III) cases [135]. Lokhandwala et al. assessed interpretive agreement between cytotechnologists and cytopathologists and found an overall Cohen’s kappa coefficient of 0.79, with the best agreement for malignant (0.91), unsatisfactory (0.89), and benign (0.83), and, although cytotechnologists tended to overcall rather undercall discrepant cases compared to cytopathologists, differences in adequacy assessment occurred in only 2% of cases, supporting the notion that cytotechnologists are well-equipped to perform ROSE for adequacy [136]. Interestingly, in a study comparing the conventional review of glass slides to scanned images of them (“virtual cytology”), intraobserver agreement was 77.5%, with a corresponding kappa value of 0.54, indicating moderate agreement between both methods, though the virtual slides were more likely to be called unsatisfactory, suggesting that such cases be reevaluated using the glass slides before sign-out [137]. Finally, a group consensus review approach was shown to minimize AUS/FLUS cases and, therefore, “could play a substantial role in the future in reducing reaspiration and/or unnecessary surgeries [138]”.

As described above, the type of follow-up used to determine the ROM varied by study for ACR TI-RADS and was different from studies of the Bethesda system, which almost uniformly relied on surgical outcomes.

## 5. Conclusions

1. Crude ROMs for ACR TI-RADS in the pediatric age group based on 1458 cases in the literature (including our cohort) were as follows:

**TR1. Benign**
ROM 2.2%
**TR2. Not Suspicious**
ROM 9.3%
**TR3. Mildly Suspicious**
ROM 16.6%
**TR4. Moderately Suspicious**
ROM 27.0%
**TR5. Highly Suspicious**
ROM 76.5%

Type of follow-up varied by study, so the ROMs for a given TR level were similarly variable. These ROMs were higher than the corresponding suggested ROMs for adults.

2. It appeared that ultrasound stratification systems performed better for PTC than FTC.
3. Perhaps the time has come to abandon size cutoffs for recommending FNA in the pediatric age group. A not insubstantial number of malignancies could be missed when pushing adult management guidelines on children and adolescents, whose thyroid glands are smaller.
4. Crude frequencies, ROMs, and RONs for the Bethesda system in the pediatric age group based on 5911 cases in the literature (including our cohort) were as follows:

**Bethesda I. Unsatisfactory**
Frequency 11.4%ROM 16.8%RON 26.7%
**Bethesda II. Benign**
Frequency 56.0%ROM 7.2%RON 27.5%
**Bethesda III. AUS**
Frequency 9.6%ROM 29.6%RON 55.8%
**Bethesda IV. FN**
Frequency 6.4%ROM 42.3%RON 86.8%
**Bethesda V. SFM**
Frequency 3.9%ROM 90.8%RON 97.6%
**Bethesda VI. Malignant**
Frequency 12.7%ROM 98.8%RON 99.7%

Follow-up was typically surgical, which was subject to partial verification bias. Nevertheless, the Bethesda categories implied higher ROMs for pediatric patients compared to adults.

5. There may be some utility in adding the ACR TI-RADS level and the Bethesda category (excluding Bethesda I) to come up with a combined score to decide whether surgery should be performed. In our cohort, there was a sharp cutoff between 7 and 8: a combined score of 7 or less had a ROM ranging from 0 to 17.6%, whereas 8 or more implied a ROM ranging from 71.4 to 100%.

## Figures and Tables

**Table 1:** TI-RADS distribution overall and ROM for our cases with surgical follow-up and combined TI-RADS/Bethesda score.

Cases Overall (208)		Mean	Mean	Cases with Surgical Follow-Up (74)			
	Distribution	Size (cm)	TI-RADS pts	Benign (41)	Malignant (33)	Total (74)	ROM
*Category*							
TI-RADS 1	8 (3.8%)	1.6	0.0	2 (2.7%)	0 (0%)	2 (2.7%)	0%
TI-RADS 2	13 (6.3%)	1.9	1.9	4 (5.4%)	1 (1.4%) 1 FTC	5 (6.8%)	20%
TI-RADS 3	56 (26.9%)	1.8	3.0	10 (13.5%)	7 (9.5%) 5 PTC, 2 FTC	17 (23.0%)	41.2%
TI-RADS 4	100 (48.1%)	1.4	4.6	20 (27.0%)	12 (16.2%) 10 PTC, 2 FTC	32 (43.2%)	37.5%
TI-RADS 5	31 (14.9%)	1.7	7.8	5 (6.8%)	13 (17.6%) 13 PTC	18 (23.0%)	72.2%
*Composition*							
Cystic	11 (5.3%)	1.6	0.8	3 (4.1%)	0 (0%)	3 (4.1%)	0%
Spongiform	3 (1.4%)	1.6	1.0	1 (1.4%)	0 (0%)	1 (1.4%)	0%
Mixed	25 (12.0%)	2.2	3.5	9 (12.2%)	1 (1.4%) 1 FTC	10 (13.5%)	10%
Solid	169 (81.3%)	1.5	4.7	28 (37.8%)	32 (43.2%) 28 PTC, 4 FTC	60 (81.1%)	53.3%
*Echogenicity*							
Anechoic	9 (4.3%)	1.8	0.3	3 (4.1%)	0 (0%)	3 (4.1%)	0%
Hyperechoic/isoechoic	84 (40.4%)	2.0	3.9	18 (24.3%)	16 (21.6%) 13 PTC, 3 FTC	34 (45.9%)	47.1%
Hypoechoic	112 (53.8%)	1.3	4.9	19 (25.7%)	16 (21.6%) 14 PTC, 2 FTC	35 (47.3%)	45.7%
Very hypoechoic	3 (1.4%)	1.3	6.7	1 (1.4%)	1 (1.4%) 1 PTC	2 (2.7%)	50%
*Shape*							
Wider-than-tall	18 (8.7%)	2.0	7.3	36 (48.6%)	28 (37.8%) 23 PTC, 5 FTC	64 (81.1%)	43.8%
Taller-than-wide	190 (91.3%)	1.6	4.0	5 (6.8%)	5 (6.8%) 5 PTC	10 (13.5%)	50%
*Margins*							
Smooth	109 (52.4%)	1.5	3.7	23 (31.1%)	13 (17.6%) 10 PTC, 3 FTC	36 (48.6%)	36.1%
Ill-defined	71 (34.1%)	1.5	4.3	11 (14.9%)	13 (17.6%) 12 PTC, 1 FTC	24 (32.4%)	54.2%
Lobulated/irregular	28 (13.5%)	2.1	6.5	7 (9.5%)	7 (9.5%) 6 PTC, 1 FTC	14 (18.9%)	50%
Extrathyroidal extension	0 (0%)	N/A	N/A	0 (0%)	0 (0%)	0 (0%)	N/A
*Echogenic foci*							
None/lg comet-tail artifacts	170 (81.7%)	1.6	3.7	35 (47.3%)	16 (21.6%) 11 PTC, 5 FTC	51 (68.9%)	31.4%
Macrocalcifications	2 (1.0%)	1.7	4.5	0 (0%)	0 (0%)	0 (0%)	N/A
Peripheral (rim) calcs	4 (1.9%)	1.4	6.3	1 (1.4%)	0 (0%)	1 (1.4%)	0%
Punctate echogenic foci	32 (15.4%)	1.8	7.5	5 (6.8%)	17 (23.0%) 17 PTC	22 (29.7%)	77.3%
**Cases with surgical follow-up (74)**				**Combined TI-RADS and Bethesda score excluding Bethesda I (69)**			
**TI-RADS points**	**ROM (Malignant/Total)**			**Benign (38)**	**Malignant (31)**	**Total (69)**	**ROM**
0	0% (0/2)		Combined score 3	1 (1.4%)	0 (0%)	1 (1.4%)	0%
1	N/A (0/0)		Combined score 4	3 (4.3%)	0 (0%)	3 (4.3%)	0%
2	20% (1/5) 1 FTC		Combined score 5	6 (8.7%)	1 (1.4%) 1 FTC	7 (10.1%)	14.3%
3	41.2% (7/17) 5 PTC, 2 FTC		Combined score 6	14 (20.3%)	3 (4.3%) 2 PTC, 1 FTC	17 (24.6%)	17.6%
4	31.3% (5/16) 4 PTC, 1 FTC		Combined score 7	11 (15.9%)	2 (2.9%) 2 FTC	13 (18.8%)	15.4%
5	0% (0/2)		Combined score 8	2 (2.9%)	5 (7.2%) 4 PTC, 1 FTC	7 (10.1%)	71.4%
6	50% (7/14) 6 PTC, 1 FTC		Combined score 9	0 (%)	4 (5.8%) 4 PTC	4 (5.8%)	100%
7	50%% (5/10) 5 PTC		Combined score 10	1 (1.4%)	6 (8.7% 6 PTC	7 (10.1%)	85.7%
8	100% (2/2) 2 PTC		Combined score 11	0 (0%)	10 (14.5%) 10 PTC	10 (14.5%)	100%
9	100% (3/3) 3 PTC						
10	100% (1/1) 1 PTC						
11	100% (1/1) 1 PTC						
12	100% (1/1) 1 PTC						

Abbreviations not introduced earlier: lg = large, calcs = calcifications, N/A = not applicable, pts = points.

**Table 2:** Risk of malignancy (Malignant/Total cases) by ACR TI-RADS category for published pediatric cohorts.

Ref.	Study Period, Location, Readers, Agreement, AUC	Age	Follow-Up	TR1 ROM (M/T)	TR2 ROM (M/T)	TR3 ROM (M/T)	TR4 ROM (M/T)	TR5 ROM (M/T)
[45]	1996–2017 Loyola University Medical Center (USA), 2 readers, intra-k = 0.69–0.77; *p* < 0.001, inter-k = 0.37; *p* < 0.002, AUC = 0.75 (95% CI, 0.64–0.86)	≤18 y	FNA or surgical	25% (1/4)	0% (0/4)	0% (0/6)	8.3% (2/24)	47.2% (17/36)
[46]	8/2007–8/2017 Children’s Hospital of Eastern Ontario (Canada), 4 readers, pairwise agreement 50.9% (95% CI, 46.3–55.5%), AUC = 0.72 (95% CI, 0.61–0.82)	<18 y	FNA, surgical, or 2+ y clinical/US stability	3.9% (0.5/12.75)	6.5% (0.75/11.5)	10% (1/10)	21.2% (6/28.25)	38% (4.75/12.5)
[47]	1/2015–2018 Aydın Adnan Menderes (Turkey), 2 readers	<18 y	Surgical *	0% (0/2)	N/A (0/0)	0% (0/4)	0% (0/2)	100% (5/5)
			Surgical or 1 y clinical/US stability	0% (0/65)	0% (0/2)	0% (0/12)	0% (0/21)	100% (5/5)
[48]	1/2004–7/2017 Brigham and Women’s and Boston Children’s Hospitals (USA), 4 readers	≤18 y	FNA or surgical; for ND FNAs, US size decrease after 1+ y or increased activity on NM scan	5.9% (2/34)	4.8% (4/83)	6.4% (7/109)	15.5% (18/116)	74.2% (46/62)
[49]	Dr. Sami Ulus Children’s Hospital (Turkey), AUC = 0.89 (95% CI, 0.80–0.98)	≤18 y	FNA or surgical	0% (0/5)	5.6% (2/36)	42.9% (3/7)	68.4% (13/19)	100% (1/1)
[6]	1/2015–3/2019 Nationwide Children’s Hospital (USA), 2 readers, inter-rater Spearman correlation and kappa statistic both 0.51; *p* < 0.00001	≤21 y	Surgical	0% (0/1)	0% (0/8)	36.4% (8/22)	66.7% (6/9)	60% (3/5)
[50]	1/2017–3/2021 University of Campania “L. Vanvitelli” (Italy), 2 readers (3rd if needed for consensus), inter-k = 0.7; *p*≤0.002	≤18 y	FNA or surgical	0% (0/4)	20% (1/5)	30% (3/10)	12.5% (2/16)	100% (6/6)
[5]	1/2000–4/2020 Asan Medical Center (South Korea), 3 readers, intra-class correlation coefficient for inter-reader agreement, 0.68 (95% CI, 0.63–0.73)	≤18 y	FNA or surgical	0% (0/11)	15.9% (11/69)	33.3% (14/42)	59.6% (31/52)	93.2% (96/103)
[51]	2007–2018 University of Pittsburgh (USA), 2 readers, weighted Cohen’s inter-k = 0.576, SE = 0.066, *p* < 0.001, AUC = 0.758	≤18 y	Surgical (91 cases) or clinical/FNA (15 cases)	0% (0/3)	25% (3/12)	36.4% (8/22)	55.2% (16/29)	80% (32/40)
[52]	2000–2020 Regina Margherita Children’s Hospital (Italy), 2 readers, Cohen’s inter-k = 0.85	<18 y	FNA (75)/surgical (40), or none	0% (0/20)	0% (0/9)	4.1% (2/49)	15.6% (17/109)	53.8% (7/13)
	**7/2015–5/2022 Phoenix Children’s Hospital (USA) ** **(current study)**	**≤18 y**	**Surgical**	**0%** **(0/2)**	**20%** **(1/5)**	**41.2%** **(7/17)**	**37.5%** **(12/32)**	**72.2%** **(13/18)**
	**Total (429/1458)**			**2.2%** **(3.5/161.75)**	**9.3%** **(22.75/244.5)**	**16.6%** **(49/295)**	**27.0%** **(123/455.25)**	**76.5%** **(230.75/301.5)**

* Not included in the statistics (the cases with either surgical or clinical US follow-up were included instead). Abbreviations not introduced earlier: CI = confidence interval, M/T = malignant/total, ND = nondiagnostic, Ref. = reference, US = ultrasound, y = years. Note that for Ref. [50], whole numbers are not provided for most of the data in parentheses; they were reported in the original article as overall number of 300 nodules (75 nodules multiplied by four readers).

**Table 3:** Frequency, risk of malignancy *, and risk of neoplasm † by Bethesda category for published pediatric cohorts.

Ref.	Study Period and Location	FNA Cases Age	Cases with Follow-Up	% Bethesda I ROM * RON †	% Bethesda II ROM * RON †	% Bethesda III ROM * RON †	% Bethesda IV ROM * RON †	% Bethesda V ROM * RON †	% Bethesda VI ROM * RON †
[53]	1/2007–7/2011 University of Pittsburgh Medical Center (USA)	179 from 142 pts ≤21 y	96 surgical	11.7% 21/179 0% 0/8 *	45.8% 82/179 6.7% 2/30 *	24.0% 43/179 28% 7/25 *	10.6% 19/179 57.8% 11/19 *	3.4% 6/179 100% 6/6 * 100% 6/6 †	4.5% 8/179 100% 8/8 * 100% 8/8 †
[54]	1/2007–1/2012 Children’s Hospital of Pittsburgh (USA)	76 ≤18 y	37 surgical	3.9% 3/76 N/A * N/A †	53.9% 41/76 44.4% 4/9 * 44.4% 4/9 †	15.8% 12/76 0% 0/8 * 0% 0/8 †	7.9% 6/76 50% 3/6 * 83.3% 5/6 †	9.2% 7/76 85.7% 6/7 * 100% 7/7 †	9.2% 7/76 100% 7/7 * 100% 7/7 †
[55]	1/2000–12/2013 Ann & Robert H. Lurie Children’s Hospital of Chicago (USA)	187 from 180 pts 177 ≤ 18 y 3 > 18 y	81 surgical	5.9% 11/187 N/A * N/A †	61.0% 114/187 10.5% 3/29 * 20.7% 6/29 †	13.9% 26/187 18.8% 3/16 * 43.8% 7/16 †	9.6% 18/187 27.7% 5/18 * 72.2% 13/18 †	3.2% 6/187 100% 6/6 * 100% 6/6 †	6.4% 12/187 100% 12/12 * 100% 12/12 †
[56]	1/1998–7/2013 Royal North Shore Children’s Hospital, Children’s Westmead Hospital (Australia)	66 from 56 pts <18 y	31 surgical	10.6% 7/66 0% 0/3 * 0% 0/3 †	57.6% 38/66 0% 0/9 * 22.2% 2/9 †	16.7% 11/66 22.2% 2/9 * 44.4% 4/9 †	6.1% 4/66 100% 4/4 * 100% 4/4 †	4.5% 3/66 100% 3/3 * 100% 3/3 †	4.5% 3/66 100% 3/3 * 100% 3/3 †
[57]	1/1998–11/2010 North Shore-Long Island Jewish Health System (USA)	282 from 282 pts <20 y	78 surgical	20.9% 59/282 10% 1/10 *	48.2% 136/282 0% 0/17 *	2.1% 6/282 50% 2/4 *	14.2% 40/282 47.4% 9/19 *	2.1% 6/282 100% 4/4 * 100% 4/4 †	12.4% 35/282 100% 13/13 * 100% 24/24 †
[58]	1995–2014 Indiana University Health, 2005–2014 University of California, Davis Medical Center (USA)	186 from 154 pts ≤18 y	61 surgical + 57 ≥ 2 y clinical	14.5% 27/186 0% 0/19 * 0% 0/19 †	61.3% 114/186 1.5% 1/68 * 8.8% 6/68 †	11.3% 21/186 26.3% 5/19 * 52.6% 10/19 †	4.3% 8/186 57.1% 4/7 * 100% 7/7 †	1.6% 3/186 100% 3/3 * 100% 3/3 †	7.0% 13/186 100% 13/13 * 100% 13/13 †
[59]	8/2010–7/2014 Ganesh Shankar Vidyarthi Memorial Medical College, Bharat Scan and Research Institute (India)	218 <18 y	44 surgical	5.5% 12/218 0% 0/2 * 0% 0/2 †	69.3% 151/218 0% 0/12 * 0% 0/12 †	10.6% 23/218 8.3% 1/12 * 75% 9/12 †	8.3% 18/218 10% 1/10 * 80% 8/10 †	2.3% 5/218 100% 2/2 * 100% 2/2 †	4.1% 9/218 100% 6/6 * 100% 6/6 †
[60]	1992–2015 The Hospital for Sick Children (Canada)	207 from 178 pts <18 y	65 surgical	26.1% 54/207 0% 0/12 * 41.7% 5/12 †	52.2% 108/207 15.8% 3/19 * 52.6% 10/19 †	8.2% 17/207 66.7% 6/9 * 77.8% 7/9 †	0% 0/207 N/A * N/A †	4.8% 10/207 71.4% 5/7 * 71.4% 5/7 †	8.7% 18/207 100% 18/18 * 100% 18/18 †
[61]	9/2008–12/2015 Connecticut Children’s Medical Center (USA)	46 from 46 pts <18 y	46 surgical	2.2% 1/46 0% 0/1 *	32.6% 15/46 0% 0/15 *	39.1% 18/46 5.6% 1/18 *	8.7% 4/46 25% 1/4 *	2.2% 1/46 100% 1/1 * 100% 1/1 †	15.2% 7/46 100% 7/7 * 100% 7/7 †
[62]	1/2001–12/2016 Agostino Gemelli Hospital of Catholic University, Loyola University (Italy, USA)	95 <19 y	95 surgical	5.3% 5/95 0% 0/5 * 60% 3/5 †	22.1% 21/95 4.8% 1/21 * 61.9% 13/21 †	9.5% 9/95 11/1% 1/9 * 88.8% 8/9 †	26.3% 25/95 20% 5/25 * 96% 24/25 †	7.4% 7/95 100% 7/7 * 100% 7/7 †	29.5% 28/95 100% 28/28 * 100% 28/28 †
[63]	2001–2018 Vanderbilt University Medical Center (USA)	302 from 253 pts ≤21 y	104 surgical	8.3% 25/302 0% 0/5 * 0% 0/5 †	71.2% 215/302 7.5% 4/53 * 20.8% 11/53 †	8.6% 26/302 20% 3/15 * 53.3% 8/15 †	3.3% 10/302 25% 2/8 * 75% 6/8% †	1.7% 5/302 100% 5/5 * 100% 5/5 †	7.0% 21/302 100% 18/18 * 100% 18/18 †
[64]	6/2003–5/2016 Istanbul University (Turkey)	103 from 80 pts ≤19 y	44 surgical	8.7% 9/103 100% 1/1 * 100% 1/1 †	49.5% 51/103 55.6% 5/9 *	11.7% 12/103 100% 3/3 * 100% 3/3 †	7.8% 8/103 71.4% 5/7 †	6.8% 7/103 85.7% 6/7 *	15.5% 16/103 100% 16/16 100% 16/16 †
[65]	1/1998–11/2016 Boston Children’s Hospital and Brigham and Women’s Hospital (USA)	430 from 334 pts <19 y	190 surgical	12.3% 53/430 30% 6/20 *	64.0% 275/430 2.6% 2/76 *	7.4% 32/430 53.8% 14/26 *	3.3% 14/430 71.4% 10/14 *	6.0% 26/430 76% 19/25 *	7.0% 30/430 100% 29/29 * 100% 29/29 †
[66]	1/2003–12/2013 Rhode Island Hospital (USA)–study only included Bethesda II FNAs	46 from 43 pts <19 y	14 surgical		N/A 14.3% 2/14 * 71.4% 10/14 †				
[67]	1/2005–5/2017 Severance Children’s Hospital (South Korea)	141 <18 y	111 surgical	6.4% 9/141 100% 2/2 * 100% 2/2 †	22.0% 31/141 12.5% 1/8 *	8.5% 12/141 75% 9/12 *	1.4% 2/141 50% ½ *	14.2% 20/141 100% 20/20 * 100% 20/20 †	47.5% 67/141 100% 67/67 * 100% 67/67 †
[68]	1/2011–9/2017 University of Michigan-Michigan Medicine (USA)	201 from 148 pts ≤20 y	100 surgical	7.0% 14/201 14.2% 1/7 * 14.2% 1/7 †	51.2% 103/201 0% 0/31 * 12.9% 4/31 †	14.9% 30/201 31.3% 5/16 * 56.3% 9/16 †	5.0% 10/201 11.1% 1/9 * 100% 9/9 †	4.5% 9/201 100% 6/6 * 100% 6/6 †	17.4% 35/201 100% 31/31 * 100% 31/31 †
[69]	2008–2018 Cincinnati Children’s Hospital (USA)	143 from 128 pts ≤22 y	74 surgical	18.9% 27/143 23.1% 3/13 *	53.8% 77/143 11.1% 3/27 *	15.4% 22/143 44.4% 8/18 *	5.6% 8/143 28.6% 2/7 *	3.5% 5/143 100% 5/5 * 100% 5/5 †	2.8% 4/143 100% 4/4 * 100% 4/4 †
[70]	12/2002–11/2018 Rady Children’s Hospital in San Diego (USA)	203 from 171 pts ≤18 y	92 surgical	14.3% 29/203 33.3% 4/12 * 41.7% 5/12 †	52.2% 106/203 26.3% 5/19 * 52.6% 10/19 †	10.8% 22/203 31.3% 5/16 * 56.3% 9/16 †	6.9% 14/203 38.5% 5/13 * 46.2% 6/13 †	3.0% 6/203 83.3% 5/6 * 83.3% 5/6 †	12.8% 26/203 100% 26/26 * 100% 26/26 †
[71]	2011–2019 7 institutions in 5 Asian countries: Japan (2), Korea (2), Thailand, Philippines, Vietnam	1217 ≤18 y	300 surgical (Philippines and Vietnam excluded)	15.9% 194/1217 30% 3/10 * 30% 3/10 †	58.3% 709/1217 8.8% 8/91 * 33.0% 30/91 †	2.6% 32/1217 66.7% 4/6 * 66.7% 4/6 †	5.5% 67/1217 36.4% 16/44 * 95.5% 42/44 †	2.3% 28/1217 100% 11/11 * 100% 11/11 †	15.3% 186/1217 99.3% 137/138 * 99.3% 137/138 †
[6]	1/2015–3/2019 Nationwide Children’s Hospital (USA)	138 from 115 pts ≤21 y		9.4% 13/138	79.0% 109/138	4.4% 6/138	1.5% 2/138	1.5% 2/138	4.4% 6/138
[72]	1/2008–12/2018 Children’s Hospital of Philadelphia (USA)	575 from 324 pts <18 y	340 surgical	4.3% 25/575 0% 0/6 *	66.4% 382/575 1.8% 3/169 *	7.8% 45/575 16.7% 7/42 *	5.7% 33/575 54.5% 18/33 *	2.3% 13/575 100% 13/13 * 100% 13/13 †	13.4% 77/575 100% 77/77 * 100% 77/77 †
[73]	2010–2021 Medical University of Lublin (Poland)	67 ≤18 y	37 surgical	4.5% 3/67 N/A *	70.1% 47/67 12.5% 2/16 *	13.4% 9/67 44.4% 4/9 *	4.5% 3/67 33.3% 1/3 *	6.0% 4/67 100% 4/4 * 100% 4/4 †	1.5% 1/67 100% 1/1 * 100% 1/1 †
[74]	2005–2020 Hospital Universitari Vall d’Hebron (Spain)	31 from 24 pts <18 y	19 surgical	25.8% 8/31 0% 0/3 * 33.3% 1/3 †	41.9% 13/31 14.3% 1/7 * 42.9% 3/7 †	12.9% 4/31 0% 0/3* 0% 0/3†	6.5% 2/31 100% 2/2 * 100% 2/2 †	0% 0/31 N/A * N/A †	12.9% 4/31 100% 4/4 * 100% 4/4 †
[75]	2000–2018 4 institutions: Portugal (1), Turkey (3)	405 from 405 pts ≤21 y	153 surgical	10.9% 44/105 30% 3/10 *	50.4% 204/405 15.2% 5/33 *	9.9% 40/405 22.2% 4/18 *	8.9% 36/105 44.4% 12/27 *	5.9% 24/105 72.7% 16/22 *	14.1% 57/405 86.0% 37/43 *
[76]	2019–2021 Children’s Hospital of Philadelphia (USA)	151 ≤19 y		2.6% 4/151	25.8% 39/151	23.2% 35/151	9.3% 14/151	4.0% 6/151	35.1% 53/151
[77]	1/2003–12/2019 Vanderbilt University Medical Center (USA)	44 ≤21 y	44 surgical	0% 0/44 N/A *	27.3% 12/44 33.3% 4/12 *	15.9% 7/44 42.9% 3/7 *	9.1% 4/44 25% 1/4	9.1% 4/44 100% 4/4 * 100% 4/4 †	38.6% 17/44 100% 17/17 * 100% 17/17 †
[78]	1/2010–10/2020 Children’s Hospital of Los Angeles (USA)	112 ≤18 y	112 surgical	4.5% 5/112 20% 1/5 *	9.8% 11/112 0% 0/11 *	26.8% 30/112 16.7% 5/30 *	11.6% 13/112 30.8% 4/13 *	15.2% 17/112 94.1% 16/17 *	32.1% 36/112 100% 36/36 * 100% 36/36 †
[79]	1/2017–5/2021 University of Alabama at Birmingham (USA)	49 ≤19 y	44 surgical + 5 clinical	4.1% 2/49 0% 0/2 *	51.0% 25/49 4% 1/25 *	14.3% 7/49 57.1% 4/7 *	8.2% 4/49 50% 2/4 *	6.1% 3/49 100% 3/3 * 100% 3/3 †	16.3% 8/49 100% 8/8 * 100% 8/8 †
	**7/2015–5/2022 Phoenix Children’s Hospital (USA)** **(current study)**	**208** **≤18 y**	**74** **surgical**	**7.7% 16/208** 40% 2/5 * **40% 2/5 †**	**56.7% 118/208** 4.8% 1/21 * **19.0% 4/21 †**	**21.6% 45/208** 27.3% 6/22 * **59.1% 13/22 †**	**2.4% 5/208** 100% 5/5 * **100% 5/5 †**	**1.4% 3/208** 100% 2/2 * **100% 2/2 †**	**10.1% 21/208** 94.7% 18/19 * **94.7% 18/19 †**
	**Total**	**5911**	**2486 surgical + ** **62 clinical**	**Freq. 11.4%** **676/5911** ROM 16.8% 27/161 * **RON 26.7%** **23/86 †**	**Freq. 56.0%** **3308/5911** **ROM 7.2%** 61/851 * **RON 27.5%** **111/403 †**	**Freq. 9.6%** **567/5911** **ROM 29.6%** 112/379 * **RON 55.8%** **91/163 †**	**Freq. 6.4%** **377/5911** **ROM 42.3%** 130/307 * **RON 86.8%** **131/151 †**	**Freq. 3.9%** **230/5911** **ROM 90.8%** **178/196** **RON 97.6%** **122/125 †**	**Freq. 12.7%** **752/5911** **ROM 98.8%** 652/660 * **RON 99.7%** **611/613 †**

**Table 4:** Accuracy measures for individual TI-RADS levels, Bethesda categories, and combined scores.

Level/Category/Score	Accuracy (%)	Sensitivity (%)	Specificity (%)	PPV (%)	NPV (%)
TI-RADS 2	47.3	97.0	7.3	45.7	75.0
TI-RADS 3	55.4	97.0	22.0	50.0	90.0
TI-RADS 4	56.8	72.7	43.9	51.1	66.7
TI-RADS 5	64.9	36.4	87.8	70.6	63.2
Bethesda II	60.8	95.5	10.0	60.9	60.0
Bethesda III	78.4	86.4	66.7	79.2	76.9
Bethesda IV	73.0	56.8	96.7	95.2	60.4
Bethesda V	66.2	45.5	96.7	95.2	54.7
Bethesda VI	63.5	40.9	96.7	94.7	52.7
Combined 4	47.8	100.0	5.3	46.3	100.0
Combined 5	55.1	100.0	18.4	50.0	100.0
Combined 6	59.4	96.8	29.0	52.6	91.7
Combined 7	73.9	87.1	63.2	65.9	85.7
Combined 8	87.0	80.7	92.1	89.3	85.4
Combined 9	82.6	64.5	97.4	95.2	77.1
Combined 10	75.4	48.4	97.4	93.8	69.8
Combined 11	68.1	29.0	100.0	100.0	63.3

## Data Availability

The data presented in this study are available upon reasonable request from the corresponding author. The data are not publicly available due to privacy.
